# Hospital Reports—Hysterical Affection of the Hip-Joint

**Published:** 1855-12

**Authors:** 


*Hospital Reports—Hysterical Affection of the Hip-Joint.* Under the care of Mr. Coulson.

The following case, the subject of which was a patient under the care of Mr. Coulson at St. Mary's Hospital, presents tolerably well-marked instance of that affection which is occasionally so troublesome to the Surgeon, known under the name of hysterical joint, supposing at least that the diagnosis made in this case was, as there is reason to believe, correct. This affection is not so frequently, perhaps, met with in Hospital practice as in private, females belonging to the richer, and consequently the more idle portions of the community being most liable to it, in common with the other affections incidental to hysterical subjects. There does not seem to be a great amount of difficulty in diagnosticating the presence of hysterical affection of a joint, if the history be duly inquired into, and the condition of the joint and limb affected carefully scrutinized. The symptoms, as is well known, are severe pain in the joint, now and then a slight puffy swelling around, together with evidence of hysteria, etc. The character of the pain is important. It is more severe than in inflammatory affections. Pressure on the joint is productive of pain when made at almost any point, and the pain produced is even greater when slight pressure is made than when a greater degree of force is used. Now it often happens that in true inflammatory disease of a joint it is found necessary to examine carefully before the surgeon finds a spot which is painful on pressure, this observation being of course intended to apply to inflammatory offections of a chronic nature, with which alone the hysterical condition is liable to be confounded. The course of the affection is peculiar, and a very important point in the diagnosis. The joint affected remains in much the same condition for a considerable time. Months and even years may elapse without any change occurring for the worse. This is hardly possible in the case of a joint in which true disease is progressively extending. One additional point observed by Mr. Coulson some time since is the fact that the limb affected is liable to frequent alternations of heat and cold, this change of temperature being not only obvious to the patient but to the observer also. In the case about to be detailed this circumstance was noticed, and together with it a condition pointed out by Sir B. Brodie, viz., a loss of sensibility of the skin covering a portion of the leg. These elements for the diagnosis of hysterical joint are of value, for their recognition may prove of great service in elucidating the nature of a case involved by particular causes in obscurity. It is singular enough that the joints most frequently presenting the hysterical affection should be also those in which chronic, inflammatory and other affections are most commonly observed.

M. A. Q., aged 24, single, by occupation a cook, was admitted into St. Mary's Hospital October 6, 1854 Her previous health is described as good, on the whole. Five years ago she had a fall, which injured the right knee to a slight extent, but of this she soon recovered, and felt no further unpleasant effects. Six weeks before her admission into the Hospital she had an attack of cholera. The catamenia commenced at a late period, at the age of 21, and have never been very regular, the period often passing by without its occurrence. Her family are tolerably healthy: one sister died of consumption. The patient is a healthy-looking young woman.

The patient ascribes her complaint to the fall on the right knee five years ago. She fancies that the right hip has been larger than the other for the last twelve months. Four months ago the right knee was swollen and painful, and leeches were applied.

*Present State.*—She is able to walk, but walks lame, and the motion of the right leg and knee gives great pain, the pain commencing at the knee, and extending up to the hip-joint. The knee is not at all swollen, but tender on pressure; there is no apparent enlargement of the parts around the hip-joint; pressure is painful all over it There is pretty constant pain in the hip-joint, and this has been present for the last three weeks. She complains of a want of feeling at the inner ankle and over the foot, on the dorsum as well as the sole. The parts thus insensible to touch are also colder to the feel than the corresponding surfaces of the other leg. This insensibility and lowering of temperature are evanescent, coming suddenly, lasting a few minutes, and then disappearing as suddenly as it came. There is no distortion, lengthening or shortening of the limb.

A belladonna plaster was applied round the hip-joint, and an ounce of quinine mixture prescribed thrice a day.

Oct. 9th.—The pain was more severe, and three grains of Dover's powder were added to each dose of the mixture. On the 11th the quinine mixture was omitted, and a combination of tincture of iron and hyoscyamus substituted.

Oct. 16th.—The hysterical and nervous condition of the patient being more completely recognized, a draught of tincture of valerian, with tincture of hyoscyamus and camphor mixture, was ordered three times a day.

Oct. 19th.—The knee is now the most painful part. The condition of the leg remains much the same. The alternate elevation and depression of temperature of the affected limb is still observed. A belladonna plaster applied to the knee.

During the remainder of the patient's stay in the Hospital matters progressively assumed a more favorable aspect. The medicines given were occasionally changed, but for the most part consisted of the remedies usually found most advantageous in hysterical cases. The patient was discharged from the Hospital November 10th, at which time she was able to walk quite readily; there was no lameness, no distortion of the limb, but the patient seemed to have a too great tendency to make much of trifling ailments.—*London Medical Times & Gazette.*

